# Expression, Purification and Initial Characterization of Functional α_1_-Microglobulin (A1M) in *Nicotiana benthamiana*

**DOI:** 10.3389/fpls.2020.593773

**Published:** 2020-12-08

**Authors:** Magnus L. R. Carlsson, Amanda Kristiansson, Jesper Bergwik, Selvaraju Kanagarajan, Leif Bülow, Bo Åkerström, Li-Hua Zhu

**Affiliations:** ^1^Department of Plant Breeding, Swedish University of Agricultural Sciences, Alnarp, Sweden; ^2^Section for Infection Medicine, Department of Clinical Sciences, Lund University, Lund, Sweden; ^3^Division of Pure and Applied Biochemistry, Department of Chemistry, Lund University, Lund, Sweden

**Keywords:** α_1_-microglobulin, *N. benthamiana*, agroinfiltration, stress protection, pharmaceutical use, heme binding

## Abstract

α_1_-Microglobulin (A1M) is a small glycoprotein that belongs to the lipocalin protein family. A major biological role of A1M is to protect cells and tissues against oxidative damage by clearing free heme and reactive oxygen species. Because of this, the protein has attracted great interest as a potential pharmaceutical candidate for treatment of acute kidney injury and preeclampsia. The aim of this study was to explore the possibility of expressing human A1M in plants through transient gene expression, as an alternative or complement to other expression systems. *E. coli*, insect and mammalian cell culture have previously been used for recombinant A1M (rA1M) or A1M production, but these systems have various drawbacks, including additional complication and expense in refolding for *E. coli*, while insect produced rA1M is heavily modified with chromophores and mammalian cell culture has been used only in analytical scale. For that purpose, we have used a viral vector (pJL-TRBO) delivered by *Agrobacterium* for expression of three modified A1M gene variants in the leaves of *N. benthamiana*. The results showed that these modified rA1M protein variants, A1M-NB1, A1M-NB2 and A1M-NB3, targeted to the cytosol, ER and extracellular space, respectively, were successfully expressed in the leaves, which was confirmed by SDS-PAGE and Western blot analysis. The cytosol accumulated A1M-NB1 was selected for further analysis, as it appeared to have a higher yield than the other variants, and was purified with a yield of ca. 50 mg/kg leaf. The purified protein had the expected structural and functional properties, displaying heme-binding capacity and capacity of protecting red blood cells against stress-induced cell death. The protein also carried bound chromophores, a characteristic feature of A1M and an indicator of a capacity to bind small molecules. The study showed that expression of the functional protein in *N. benthamiana* may be an attractive alternative for production of rA1M for pharmaceutical purposes and a basis for future research on A1M structure and function.

## Introduction

α_1_-Microglobulin (A1M) is a small glycoprotein with a molecular mass of about 26 kDa (Ekström and Berggård, 1977), consisting of 183 amino acids (Kaumeyer et al., 1986), and featuring one O-linked and two N-linked glycosylation sites (Amoresano et al., 2000) as well as a single disulfide bond between the Cys72 and Cys179 residues (Mendez et al., 1982). It is a member of the lipocalin protein family (Flower, 1996), displaying the characteristic lipocalin tertiary structure: an eight-stranded β-barrel with a closed bottom and an open end exposing a ligand-binding pocket (Meining and Skerra, 2012). A1M is present in a wide range of vertebrate species (Olsson et al., 2012) and a wide range of tissues (Åkerström and Gram, 2014), though at particularly high levels in the liver, blood plasma and kidney (Åkerström, 1983). Its main site of expression is the liver (Tejler et al., 1978) from where it is secreted into the blood, after which it can be extraverted into other tissues or quickly removed by the kidney through glomerular filtration (Wester et al., 2000; Larsson et al., 2001). In the kidney, it is reabsorbed and degraded in the tubules, with a minor fraction passed into the urine (Nordberg et al., 2007). An interesting property of the protein is its yellow-brown color, reportedly caused by heterogeneous covalent modifications of various amino acid sidechains (including Lys92, Lys118, Lys130, and Cys34), resulting in the generation of “chromophores” (Escribano et al., 1991; Berggård et al., 1999), which have been suggested to be the cause of the observed size and charge heterogeneity of A1M (Åkerstrom et al., 2000). A1M is also known to interact and form complexes with other plasma proteins, notably IgA (Grubb et al., 1983; Berggård et al., 1997).

During recent years, an understanding of the biological role of A1M has emerged, describing it as a reductase and a heme- and radical-binding protein (Allhorn et al., 2002, 2005; Åkerström et al., 2007; Olsson et al., 2012; Åkerström and Gram, 2014). Based on these properties, A1M has been shown to protect cells and tissues against heme, reactive oxygen species (ROS), and radiation-induced oxidative damage *in vitro* and *in vivo* (Olsson et al., 2008, 2011; Kristiansson et al., 2019, 2020a,b). As an antioxidant with protective properties, the protein has also attracted interest as a pharmaceutical candidate. Preeclampsia and acute kidney injury during coronary artery bypass grafting surgery have been identified as possible target conditions, since these are associated with both oxidative stress and elevated levels of free hemoglobin/heme (Gunnarsson et al., 2017). In a recent review, Kristiansson et al. (2020c) discussed the *in vitro* and *in vivo* data available for A1M and additional medical conditions where investigation of A1M as a future treatment could be of interest, including certain anemias, intraventricular hemorrhage in preterm infants, complications of blood transfusions and atherosclerosis. Another possible medical application of A1M could be co-administration with other pharmaceuticals as a means to control toxicity, for example hemoglobin-based oxygen carriers (HBOC’s), as the toxicity of free heme and uncontrolled oxidative processes can be a concern for that class of drugs, which is intended as a complement or alternative to donated blood (Alayash, 2014).

Production of rA1M for pharmaceutical purposes using *E. coli* has previously been reported [appr. 30–40 mg/L, purified A1M-WT and A1M-035 (with N17D, N96D, R66H mutations)] (Kwasek et al., 2007; Åkerström et al., 2019). *E. coli* produced, his-tagged, rA1M variants were in these studies refolded from inclusion bodies, after which they appeared to be correctly folded and contained a disulfide bond. Interestingly, *E. coli* produced rA1M displays both an increased redox activity compared to native A1M isolated from plasma or urine and a reduced level of chromophore (Allhorn et al., 2005). Moreover, the amino acids associated with covalent modifications (Escribano et al., 1991; Berggård et al., 1999) are also connected to A1M activity (Allhorn et al., 2005; Åkerström et al., 2007), and the formation of covalent modifications may be linked to A1M’s radical binding mechanism (Åkerström et al., 2007; Åkerström and Gram, 2014). Saturation of these amino acids with chromophore might therefore represent depletion of the radical binding capacity, leading to a reduction in activity. One major drawback with the use of *E. coli* produced rA1M for pharmaceutical purposes has been suggested to be a limited solubility and stability, which leads to aggregation, and poses various practical challenges for pharmaceutical use (Åkerström et al., 2019). This has been suggested to likely be a result of the lack of glycosylation, and for the modified A1M-035 variant, amino acid substitutions at sites corresponding to the glycosylation sites of native A1M (i.e., N17D and N96D) in combination with a R66H substitution, has been shown to improve these properties (Åkerström et al., 2019). In addition to *E. coli*, production of rA1M has also been reported using baculovirus infected insect cells (10 mg scale, 21 mg/L purified A1M) (Wester et al., 1997), while A1M has been produced in analytical scale using mammalian liver cell lines (HepG2, ∼0.6 mg/L) (Åkerström et al., 1995). These studies did not focus on medical use however and protective activity was not examined. Similarly to native A1M, A1M from mammalian and rA1M from insect cell culture displayed a higher chromophore level, compared to *E. coli* produced one (Kwasek et al., 2007). The insect cell produced rA1Ms carried a higher chromophore level than plasma A1M (Wester et al., 1997), and may, therefore have a disadvantage in this regard.

In light of the potential medicinal interest in the protein, additional suitable expression systems could be of interest. Particularly as expression of rA1M in other systems suffer from various drawbacks, i.e., *E. coli*-produced rA1M needs to be refolded from inclusion bodies which complicates the process, lowering yield and increasing the cost, while insect cell rA1M is heavily modified with chromophores, and mammalian cell expression has only been carried out in analytical scale, as discussed in the previous section. Plants have several advantages that make them interesting for production of protein pharmaceuticals. They can, for example, be cultivated easily in large scale in contained greenhouses, have capacity for complex glycosylation (Strasser, 2016), and lack immunogenic bacterial endotoxins, which pose an additional purification challenge for *E. coli*–produced proteins (Magalhaes et al., 2007). The principal strategies for recombinant protein expression in plants are nuclear and chloroplast transformation, and transient expression (Paul and Ma, 2011). The first two strategies involve stable integration of genes of interest in the plant genome and generation of transgenic plants expressing the proteins of interest, using *Agrobacterium tumefaciens* or biolistic particle bombardment. In contrast, the transient expression strategy relies on temporary gene expression in plant cells rather than the generation of transgenic plants (Paul and Ma, 2011; Chen et al., 2013). The transient gene expression strategy often relies on treatment of adult plants with *A. tumefaciens* carrying viral vectors and has been developed as an alternative to stable transformation for molecular farming purposes due to high yields and faster development times (Chen et al., 2013; Yao et al., 2015). The tobacco mosaic virus based pJL-TRBO is one example of this type of vectors, and has been shown to express 3–5 mg green fluorescent protein (GFP) per g fresh weight leaf in one study (Lindbo, 2007b), with comparable values (1–5 mg/g range) reported for several other similar transient expression vectors (Marillonnet et al., 2005; Sainsbury et al., 2009; Diamos et al., 2020). Relatively high yields are also possible with stable transformation (up to 0.5 g/kg nuclear transformation, 5 g/kg chloroplast transformation) (Schillberg et al., 2019), although these high expression levels may be exceptions as degree of accumulation also depends on the specific protein and other factors, regardless of expression strategy (see Table in Shanmugaraj et al., 2020, for some example yields).

Moreover, expression of A1M in plants might also reveal new information concerning the properties of the protein. Both glycosylation and chromophore formation appear to differ between expression systems, such as between insect or mammalian cell culture (Åkerström et al., 1995; Wester et al., 2000) or *E. coli* (Kwasek et al., 2007). Data from additional expression systems could perhaps provide new information concerning compounds and mechanisms involved in chromophore formation.

In this study, we aimed at exploring the possibility of producing functional rA1M in the leaves of *Nicotiana benthamiana* by transient expression using the high expression pJL-TRBO viral vector (Lindbo, 2007b). Our results show that the plant production system was capable of producing a functional rA1M protein, with mutations based on the A1M-035 variant, in the cytosol. Two glycosylated variants were also expressed but were not further tested for functionality in this study.

## Materials and Methods

### Plant Material

The seeds of *N. benthamiana* were sown in standard planting soil with no extra fertilization and grown in small pots for 2 weeks. Thereafter, the seedlings were transplanted to 2 L pots, with one plant in each, and grown for another 3–4 weeks before agroinfiltration. The plants were grown in a climate controlled chamber at 60% relative humidity with cycles of 18 h light (25°C, 250 μmol m^–1^ s^–1^) and 6 h dark (20°C).

### Vector Construct Design

Three vector constructs were designed in order to test the expression of rA1M variants in the cytosol as well as when targeted to the secretory pathway. The 183 amino acid A1M sequence (Kaumeyer et al., 1986; Åkerström et al., 2019) was used as a base and elements were added to it as described below. All the constructs included a leading Kozak consensus sequence and the required stop codons and flanking restriction enzyme sites (PACI and XMAJI). An N-terminal extension consisting of a His-tag and an enterokinase cleavage site [(M)HHHHHHHHDDDDK] was also included for all constructs to facilitate purification and improve solubility (Allhorn et al., 2005; Kwasek et al., 2007; Åkerström et al., 2019). The first construct, hereafter termed *A1M-NB1*, was intended for cytosolic accumulation and included the amino acid substitutions N17D and N96D in the A1M sequence for increased stability, as described by Åkerström et al. (2019) for the A1M-035 variant. In addition to this, two more constructs, *A1M-NB2* and *A1M-NB3*, were designed for endoplasmic reticulum (ER) accumulation and secretion of the expressed proteins, respectively. These two constructs were equipped with an N-terminal signal peptide sequence (from tobacco PR-1a, Uniprot accession nr. P08299; The UniProt Consortium, 2019), which has been used for extracellular localization (Dixon et al., 1991). *A1M-NB2* also carried a C-terminal ER retention peptide (KDEL) connected by a minimal linker (GS). The designed vectors were synthesized by Thermo Fisher GeneArt Service (Waltham, MA, United States), whose codon optimization tool was used. The sequences used in the study are available in the Supplementary Data.

### *E. coli* Recombinant α_1_-Microglobulin

The wildtype rA1M (A1M-WT) and the mutated rA1M-variant A1M-035, carrying the amino acid substitutions N17D, R66H, and N96D, were previously expressed in *E. coli*, and were refolded from solubilized inclusion bodies and purified as described by Åkerström et al. (2019).

### Molecular Cloning

In this study, the viral vector pJL-TRBO (Lindbo, 2007b) was used for transient expression. *Pac I* and *Xma JI* (Avr II) fast digest restriction enzymes (Thermo Fisher Scientific) were used to cleave the vector and the ordered rA1M sequences, which were then isolated and retrieved using gel electrophoresis and a gel extraction kit (Thermo Fisher Scientific). The rA1M sequences were then ligated into the cleaved vector and transformed into *E. coli* competent cells (HST08, Stellar competent cells) (Takara Bio, Kusatsu, Japan), and grown on kanamycin selection medium. Colony PCR, with vector specific primers (Supplementary Data), was used to screen for transformation events and selected colonies were cultivated in liquid Luria Berthani (LB) medium with kanamycin for plasmid isolation, for which a plasmid miniprep kit was used (Thermo Fisher Scientific). The isolated plasmids were sequenced by Eurofins genomics (Ebersberg, Germany) before electrotransformation into competent cells of *A. tumefaciens* strain GV3101:pMP90. Colony PCR was again used for screening.

### Agroinfiltration

*Agrobacterium* inoculation suspensions were prepared as previously described (Lindbo, 2007b). *Agrobacterium* suspensions containing the pJL-TRBO-rA1M with an OD_600_ of 1.0 were mixed in a 2:1 ratio with inoculation suspension containing the pJL3-p19 vector (Lindbo, 2007a) and injected into the *N. benthamiana* leaves. The leaves were harvested 4 days after infiltration and were immediately frozen at −80°C for further use.

### Protein Extraction and Purification

Harvested leaves were cryogrinded in an RM200 mortar grinder (Retsch, Haan, Germany). Soluble protein was extracted using 3 parts (volume/mass) of extraction buffer [100 mM Tris-HCl pH 8.5, 10% glycerol, 0.5 M NaCl, 20 mM imidazole, 5% polyvinylpolypyrrolidone (PVPP), 5 mM dithiothreitol (DTT), 0.1% plant protease inhibitor cocktail (PI) (P9599) (Sigma-Aldrich, St. Louis, United States)]. Centrifugation (∼18,000 RCF, 4°C, at least 30 min followed by ∼38,000 RCF, 4°C, at least 20 min for A1M-NB1, ∼18,000 RCF 4°C, at least 30 min for A1M-NB2 and A1M-NB3) and filtration using a food grade polyamide mesh were used to remove solid material. For the protein purification, the extraction was followed by filtration using 0.2 μm sterile filters and addition of Tween 20 at ∼0.1%. Filtration with 0.2 μm sterile filters was repeated as required and the protein extract was then loaded, with a hand-operated syringe, on 1 or 5 mL Histrap IMAC columns (GE healthcare, Chicago, United States). The columns, precharged with Ni-ions, were equilibrated with binding buffer (50 mM Tris-HCl pH 8, 0.5 M NaCl, 10% glycerol, 20 mM imidazole, 5 mM DTT, 0.1% PI) prior to loading. After loading the extracts, the column was washed with 5–10 column volumes (CV) of binding buffer followed by a second wash with 5–10 CV binding buffer without DTT and PI. Elution was done with buffer containing 50 mM Tris-HCl pH 8, 0.5 M NaCl, 0.5 M imidazole, added stepwise or as a steep gradient, at a 5 mL/min flow rate using a BioLogic LP System (Biorad, Hercules, CA, United States). A1M-NB2 and A1M-NB3, intended for the deglycosylation test described below, were not further purified at this point, but were buffer changed to 20 mM Tris-HCl buffer, pH 8.0, 150 mM NaCl using repeated dilutions and 5 or 10 kDa Vivaspin concentrators (Sartorius, Göttingen, Germany). The recovered A1M-NB1 fractions were buffer-changed to ion exchange (IEX) binding buffer (20 mM Tris-HCl, pH 8), using a 5 kDa Vivaspin concentrator (Sartorius) and repeated dilutions, with 1 mM ethylenediaminetetraacetic acid (EDTA) included in the first dilution. The material was then purified using a 5 mL Hitrap Q-Sepharose column (GE healthcare), equilibrated with IEX binding buffer. After loading of the sample, the Q-Sepharose column was washed with 5–10 CV IEX binding buffer (20 mM Tris-HCl pH 8) and the sample was eluted using a 0–350 mM NaCl gradient over 20 CV. A flow rate of 5 mL/min was used. The collected fractions were then concentrated using 5 kDa molecular weight cut-off (MWCO) Vivaspin concentrators to ∼20 mg/mL (∼2 mL) and purified with size exclusion chromatography using a preparative column of 160 mL Sephacryl S200. Elution was done with 20 mM Tris-HCl buffer, pH 8.0, 150 mM NaCl at a flow rate of 8 mL/h. Elution fractions of ∼2 mL were collected. Eluted fractions were subjected to absorbance spectroscopy and sodium dodecyl sulfate–polyacrylamide gel electrophoresis (SDS-PAGE) analysis, and fractions of high purity, with no aggregates and minimal dimer were selected and pooled. A fraction of the purified protein was further treated using the Pierce endotoxin removal kit (Thermo Fisher Scientific), according to the manufacturer’s instruction, for use in cell protection tests, as an additional precaution. For adjusting buffers for protein analysis Sephadex G25 PD10 columns (GE healthcare) and 5 or 10 kDa MWCO Vivaspin concentrators (Sartorius) were used. Extraction and purification were performed at 4°C, or on ice, whenever possible. The sample was stored overnight between the IMAC, IEX and size exclusion chromatography steps (SEC). The purified samples were flash frozen in liquid N_2_ and stored at −80°C.

### SDS-PAGE and Western Blot Analyses

SDS-PAGE and Western blot analyses were used to evaluate the purity and confirm the identity of the plant produced rA1M proteins. The protein samples were prepared by mixing with 4x Bolt LDS sample buffer (Thermo Fisher Scientific) and 10x Bolt sample reducing agent (Thermo Fisher Scientific) (omitted for non-reduced samples) and H_2_O in the recommended proportions, followed by heating at 70°C for at least 10 min. The prepared samples were then loaded in Bolt 4–12% Bis-Tris Plus gels which were run at 145 V for 45 min. The gels not intended for Western blotting were then washed in MilliQ water 3 times and stained with SimplyBlue^TM^ SafeStain (Thermo Fisher Scientific) for ∼1 h before destaining in MilliQ water. For Western blotting the gels were briefly washed in MilliQ water after which the proteins were transferred to PVDF membranes using iBlot 2 Transfer Stacks and an iBlot^TM^ 2 Gel Transfer Device (1 min at 20 V, 4 min at 23 V, 2 min at 25 V) and washed in MilliQ water. The membranes were treated for 1 h at RT with SuperBlock^TM^ (PBS) Blocking Buffer (Thermo Fisher Scientific), which was also used for diluting the antibodies. The membranes were then treated with 500x dil. rabbit anti-6xHis-tag polyclonal primary antibody (Thermo Fisher Scientific, cat. PA1-983B) for ∼30 min, followed by four washes in PBS-Tween 20, ∼5 min each, and then treated with 10,000x dil. Goat anti-Rabbit IgG (H+L) Secondary Antibody, HRP (Thermo Fisher Scientific, cat. A16096) for ∼30 min and similarly washed. The membranes were then briefly washed in MilliQ water, treated using Novex^TM^ ECL Chemiluminescent Substrate Reagent Kit (Thermo Fisher Scientific) according to manufacturer’s instructions, and analyzed with a ChemiDoc MP Imaging System (Bio-Rad, Hercules, CA, United States).

### Determination of Absorbance Spectra

The absorbance spectra of A1M-NB1 and *E. coli* produced A1M-WT (as a reference) were determined using a MultiSkan GO spectrophotometer (Thermo Fisher Scientific). An extinction coefficient of 34,045 M^–1^ cm^–1^ at 280 nm and a molecular mass of 22,435.23 g/mol was calculated with the ProtParam online tool (Gasteiger et al., 2005) and used for quantification. A NanoDrop 1000 instrument (Thermo Fisher Scientific. Saveen Werner is a distributer) was also used for protein quantification during sample preparations. For presentation, some absorbance values were presented as normalized values, i.e., ratio of the max 280 nm peak value to compare variations in chromophore.

### Analysis by Fluorescence Spectroscopy

Fluorescence analysis was performed with a Jasco J-810 spectrofluorimeter equipped with an FMO-427 monochromator and using a 100 μL quartz cuvette (Hellma Precision Cell, type 105.251-QS, light path length 3 mm in both excitation and emission modes) under nitrogen flow. Temperature was held at 21°C. Tryptophan fluorescence of 50 μg/mL A1M-NB1 or *E. coli* produced A1M-WT in 20 mM Tris-HCl, 150 mM NaCl, pH 8.0 was measured by exciting at 290 nm and reading emission between 320 and 400 nm with slit width 5 nm. Emission spectra were recorded at 1 nm intervals 5 times and an average spectrum was calculated. At these conditions, the fluorescence signals of the proteins were well resolved within the detector sensitivity at 800 V. Chromophore fluorescence of rA1M protein at 0.81 mg/mL dissolved in 20 mM Tris-HCl, 150 mM NaCl, pH 8.0 was measured by exciting at 350 nm and reading emission between 400 and 550 nm with slit width 5 nm. Emission spectra were recorded at 1 nm intervals 5 times and an average spectrum was calculated, setting detector amplification at 900 V.

### Biophysical Properties Tests

Temperature stability of the A1M-NB1 protein was analyzed on a Prometheus NT.48 (NanoTemper technologies, Munich, Germany) differential scanning fluorimetry (DSF) instrument. A1M-NB1, or *E. coli*-produced A1M-WT protein at ∼0.4 mg/mL dissolved in PBS adjusted to pH 8.0, was analyzed, using a temperature increase of 1°C/min over a range from 20 to 95°C and 50% excitation power. Hydrodynamic radius was measured under the same buffer conditions at 20°C using a Zetasizer APS DLS instrument (Malvern Panalytical, Almelo, Netherlands). The radius was determined as an average from six reads based on the main observed peak (the largest peak in terms of relative calculated mass) of each measurement. Ellman’s test (Ellman, 1959) was used to determine the number of free sulfhydryl groups per mol of A1M-NB1 under non-denaturing conditions. In this test, the difference in absorbance at 412 nm, after diluting the rA1M samples 1:10 in reaction buffer (100 mM sodium phosphate buffer, pH 8.0, 1 mM EDTA) with or without 5,5-dithio-bis-(2-nitrobenzoic acid (DTNB) (80 μg/mL) and incubating for 15 min, was measured for a known concentration of A1M-NB1 (0.117 mg/mL). An extinction coefficient of 14.15 mM^–1^cm^–1^ at 412 nm for the product (Riddles et al., 1983) was used. In addition to buffer blank, DTNB (start conc.) and protein (chromophore) absorbances were measured separately and subtracted.

### Deglycosylation Test

Glycosylation status of the plant produced rA1M variants (A1M-NB1, A1M-NB2, A1M-NB3) was tested using PNGase F Glycan Cleavage Kit (Thermo Fisher Scientific, cat. A39245). About 2.3 μg of each rA1M protein or the ovalbumin control sample (Sigma-Aldrich, cat. A5503) was diluted to 12 μL with dH_2_O and 1 μL 5% SDS with 1 μL 1M DTT (1μL dH_2_O for non-reduced samples). The samples were then denatured by heating at 95°C for 5 min, followed by cooling at RT for 5 min. After addition of 2 μL PNGase reaction buffer (Thermo Fisher Scientific, cat. A39245), 2 μL 10% triton X-100 and 2 μL PNGase F (Thermo Fisher Scientific, cat. A39245), the sample was incubated for 2 h at 37°C. The reactions were then analyzed by SDS-PAGE as described in the section above, all 20 μL of the deglycosylation reactions for each sample was loaded on the gel.

### Heme Binding Test

Heme binding was analyzed as previously described (Karnaukhova et al., 2014). A stock solution of heme was prepared by dissolving hemin in dimethyl sulfoxide (DMSO) to 20 mM, followed by further dilution to 250 μM in Tris-buffer, pH 8.0. For the test, 15 μg A1M-NB1 or *E. coli* produced A1M-WT was incubated with heme (heme:rA1M molar ratio range 0–4) in Tris-buffer, pH 8.0, for 30 min at RT. The protein samples were then separated by native PAGE on stain-free 12% Criterion TGX gels (Bio-Rad) at 150 V for 60 min. The bands on the gels were analyzed using a ChemiDoc MP instrument (Bio-Rad) for tryptophan fluorescence using the stain-free setting, followed by staining with Coomassie Brilliant Blue and destaining with MilliQ water before imaging on the ChemiDoc MP using the Coomassie setting. Both sets of bands were quantified using Image Lab Software (Bio-Rad). The heme binding was estimated as the amount of quenching of the tryptophan fluorescence relative to the bands of the non-heme-treated rA1M. The migration of the bands was determined by measuring the distance from the edge of the gel to each band. The relative migration was calculated for each sample by comparing it to the rA1M sample without heme.

In another test, absorption spectroscopy was used to detect the spectra of the heme-A1M-NB1 complex. For this purpose, hemin (Sigma-Aldrich, cat. 51280) was dissolved in DMSO and adjusted to 4 mM, based on its extinction coefficient at 404 nm of 183 mM^–1^ cm^–1^ (Collier et al., 1979). Reaction mixtures of ∼0.1 mg/mL A1M-NB1 and various heme concentrations (heme:rA1M molar ratio 2.5, 2, 1.5, 1, 0.75, 0.5, and 0.25) were prepared in 20 mM Tris-HCl buffer, pH 8.0, 150 mM NaCl. DMSO stock was prediluted in the same buffer immediately before preparation of the reaction mixtures, to minimize DMSO concentration (<0.3%). The reaction mixtures were incubated at RT for ∼3 h before analysis on a MultiSkan GO spectrophotometer.

### Cell Protection Assay

The capacity of rA1M-NB1 to protect red blood cells (RBC) against spontaneous, heme-induced or osmosis-induced hemolysis was estimated essentially as described (Kristiansson et al., 2020a). Blood was drawn from healthy voluntary donors (Ethical Review Board in Lund: Permission no. 2015/801) in K_2_EDTA-coated vacutainers (BD). The blood was transferred to a Falcon tube and centrifuged (800 × *g*, 10 min) for blood fractionation. The RBCs were washed 5 times with PBS (pH 7.4) and diluted in PBS to 1% suspension (v/v). Thereafter, RBCs were exposed to stress in 1.5 mL Eppendorf tubes as follows. For spontaneous hemolysis, RBCs were incubated at room temperature with or without addition of A1M-NB1 or *E. coli* produced A1M-WT (220 μg/mL). For heme-induced hemolysis, RBCs were incubated in PBS at room temperature with 30 μM heme, with or without A1M-NB1 or *E. coli* produced A1M-WT (220 μg/mL). The heme was added from a 1 mM stock solution after dissolving heme in 0.1 M sodium hydroxide and diluting with PBS. For osmosis-induced hemolysis, RBCs were incubated at room-temperature with 45% MilliQ water, with or without A1M-NB1 or *E. coli* produced A1M-WT (220 μg/mL). After 2 h of incubation (end-over-end rotation, 8 rpm, RT) the tubes were centrifuged (500 × *g*, 5 min) and the supernatants were analyzed for content of lactate dehydrogenase (LDH) released from ruptured RBC. The LDH release was measured in the supernatants with CytoTox 96 Non-Radio Cytotoxicity Assay (Promega) according to manufacturer’s instructions. The absorbance was measured at 490 nm using a VICTOR 1420 multilabel plate reader (PerkinElmer, Waltham, MA, United States).

## Results

### rA1M Protein Expression, Purification and Initial Analysis

Three modified rA1M variants A1M-NB1, A1M-NB2, and A1M-NB3, designed for localization to the cytosol, ER or extracellular space, respectively, were used to test plant expression of rA1M in this study. The three proteins carried a His-tag to facilitate purification. A1M-NB1 also carried two mutations (N17D, N96D) for stability/solubility, based on the A1M-035 mutant (Åkerström et al., 2019). The R66H mutation of A1M-035 was omitted in this construct, as preliminary, unpublished results indicated that the impact of that mutation on these properties was marginal, and we preferred to minimize the difference with the native sequence. Following molecular cloning and transformation procedures, agroinfiltration (*Agrobacterium* strain GV3101) was used to deliver the pJL-TRBO vectors, carrying either the *A1M-NB1*, *A1M-NB2* or *A1M-NB3*, into the plant cells for expression.

**FIGURE 1:**
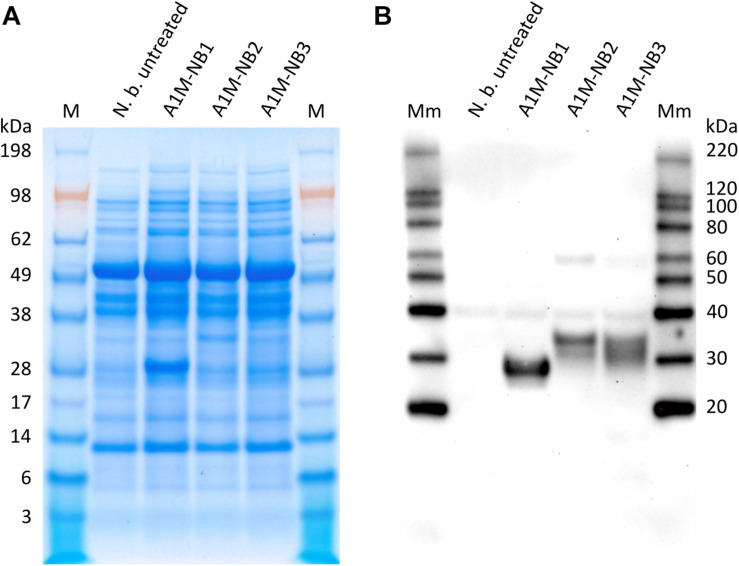
SDS-PAGE **(A)** and corresponding Western blot (anti-His-tag) **(B)** of the protein extracts from untreated control *N. benthamiana* (N. b. untreated) and of extracts from *N. benthamiana* agroinfiltrated with the three rA1M constructs (*A1M-NB1, A1M-NB2, A1M-NB3*, designed for cytosol, ER-accumulation and secretion, respectively). Production of the corresponding rA1M proteins were confirmed in the Western blot and with SDS-PAGE for A1M-NB1. 10 μL extract was loaded per well. M, Seeblueplus2 protein standard; Mm, MagicMark XP Western protein standard.

Following the agroinfiltration procedure, SDS-PAGE and Western blot were used to analyze the extracts obtained from the treated *N. benthamiana* leaves, in order to confirm expression of the three rA1M proteins (Figure 1). A1M-NB1 appeared to be expressed at a higher level than the ER- and extracellular targeted variants, A1M-NB2, and A1M-NB3, when observed visually on Coomassie stained SDS-PAGE (Figure 1A), with A1M-NB3 only clearly detectable in Western blot (Figure 1B). Due to the lack of clearly defined bands in SDS-PAGE and as the Western blot was not set up for quantitative comparison, which would probably be complicated by the “smearing” of bands, further quantitative comparison using image analysis was not considered meaningful. A1M-NB1 had an apparent size of approximately 25–30 kDa, which is similar to *E. coli*-expressed rA1M (Kwasek et al., 2007), but slightly higher than the theoretical molecular mass, 22.5 kDa. Although of roughly the same theoretical peptide molecular mass, given correct tag cleavage, A1M-NB2 and A1M-NB3 had larger apparent sizes, and appeared to be more heterogeneous, with A1M-NB3 in particular appearing as a smear, rather than a distinct band (Figure 1B). As the two variants were expected to be glycosylated, the increased size and heterogeneity were most likely results of this. As the initial SDS-PAGE analysis suggested a higher yield and possibly a higher degree of homogeneity for A1M-NB1 and due to the potential challenges for the pharmaceutical use of glycosylated rA1M, we decided to focus on the A1M-NB1 variant for further functional analysis in this study.

**FIGURE 2:**
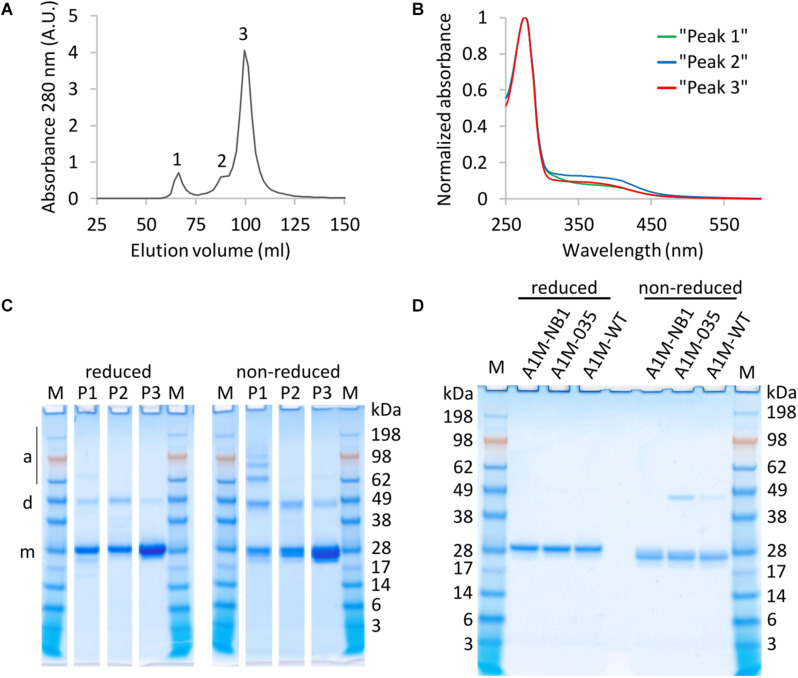
Absorbance and SDS-PAGE analysis of SEC fractions and SDS-PAGE of final purified and pooled material. **(A)** Chromatogram, showing three peaks of eluted protein **(B)** Normalized absorbance spectra (ratio of max absorbance) of a fraction from peak 1 (green), peak 2 (blue), and peak 3 (red), showing variation in chromophore absorbance. **(C)** SDS-PAGE of SEC fractions from the peaks, reduced and non-reduced. Band positions of putative monomer (m), dimer (d) and aggregate/multimer (a) are indicated. 6 μL sample was loaded for P1 and P2, 3 μl was loaded for P3. **(D)** SDS-PAGE (reduced and non-reduced) showing purified A1M-NB1 from pooled “peak 3” fractions, and two *E. coli* produced protein samples for comparison (A1M-035 and A1M-WT). ∼2 μg was loaded of each sample. M, Seeblueplus2 protein standard.

A1M-NB1 was purified by IMAC, IEX, and SEC. The final SEC step showed three main peaks in its chromatogram, likely corresponding to aggregated-, dimeric- and monomeric A1M-NB1 (Figure 2A), with slight variation in the absorbance spectrum, suggesting variation in amount of chromophore (Figure 2B). SDS-PAGE under reducing and non-reducing conditions was used for further analysis (Figures 2C,D). Aggregated forms as well as monomeric A1M-NB1 were seen in the high-molecular mass peak, and putative dimers were present in all peaks. Smaller amounts of the aggregated forms and the dimer band were seen under reducing conditions, probably indicating that these are partially cysteine mediated (Figure 2C). SEC fractions with high purity and low levels of dimers, as indicated by visual observation on SDS-PAGE (SDS-PAGE of selected fractions shown in Figure 2C) were pooled and the final, highly purified (Figure 2D), A1M-NB1 protein was used for further analyses.

One structural feature of native A1M is a disulfide bond between the Cys72 and Cys179 residues (Mendez et al., 1982). The cysteine bridge is also present in *E. coli* produced rA1M, following *in vitro* refolding (Åkerström et al., 2019). To evaluate whether the disulfide bridge was present, A1M-NB1 was compared with two *E. coli* produced rA1Ms (A1M-035 and A1M-WT) on reducing and non-reducing SDS-PAGE (Figure 2D). The plant produced A1M-NB1 displayed a similar size as the bacterially produced rA1Ms (Figure 2D). There was also a clear reduction in migration speed (around 5 kDa increase in apparent size) of the A1M-NB1 protein under reducing conditions, compared to non-reducing, similarly to the bacterially produced rA1Ms, suggesting that the majority of the purified protein probably has an internal disulfide bridge and a free Cys34. A1M-NB1 displayed some variation in the non-reduced state in some other gels however, with a somewhat broader or double band (Figure 2C). A deglycosylation test using PNGase F was also performed to see if the (IMAC purified) A1M-NB2 and A1M-NB3 carried the expected N-glycosylation (Supplementary Figures 1A,B). The results showed a full deglycosylation of the A1M-NB2 variant, as a single band of similar size as the A1M-NB1 control was seen. The A1M-NB3 instead appeared to be partially deglycosylated, as a band of similar size as A1M-NB1 was accompanied by a higher molecular mass smear. The deglycosylation was performed both under reducing and non-reducing conditions and the A1M-NB2 and A1M-NB3 displayed a similar gel mobility shift as the A1M-NB1 between these conditions (Supplementary Figure 1B).

The yield of A1M-NB1 was estimated from the 280 nm absorbance peak as ∼50 mg purified A1M-NB1 per kg leaf (fresh weight), after the three protein purification steps. The calculated extinction coefficient of 34,045 M^–1^cm^–1^ (Gasteiger et al., 2005) was used.

### Spectroscopic Analysis

**FIGURE 3:**
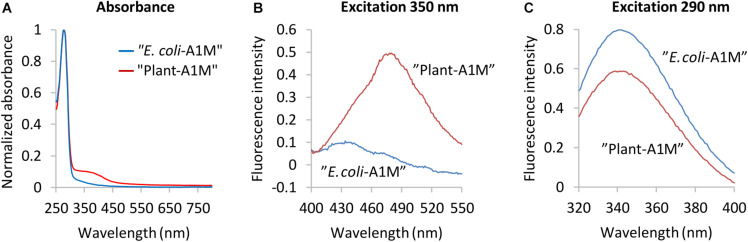
Spectroscopic analyses of *E. coli* produced A1M-WT and plant-produced A1M-NB1. **(A)** Spectroscopic analyses of *E. coli* produced A1M-WT and plant-produced A1M-NB1. **(A)** Absorbance spectra (normalized, ratio of max absorbance) of plant produced A1M-NB1 (red) and *E. coli* produced A1M-WT (blue). **(B)** Chromophore fluorescence of 0.81 mg/mL plant-produced A1M-NB1 (red) and *E. coli*-produced A1M-WT (blue), excitation wavelength 350 nm. **(C)** Tryptophan fluorescence spectra of 50 μg/mL plant-produced A1M-NB1 (red) and *E. coli*-produced A1M-WT (blue), excitation wavelength 290 nm. The fluorescence spectra **(B,C)** show averages of five measurements.

Visually, the purified rA1M (NB1, NB2, NB3) displayed a yellow-brown color previously shown to be associated with A1M and its chromophore (Ekström and Berggård, 1977; Åkerstrom et al., 2000). The absorbance spectra of purified A1M-NB1 (Figures 2B, 3A) and partially purified A1M-NB2 and A1M-NB3 (Supplementary Figure 2) reflected this, with an increased absorbance between 300 and 450 nm gradually decreasing at longer wavelengths (Figures 2B, 3A). A1M-NB1 displayed a more pronounced absorbance increase in this region than seen in the *E. coli* A1M-WT samples, as well as a shoulder around 390 nm, which is not seen for the bacterial A1M-WT (Figure 3A) (Kwasek et al., 2007). The plant produced A1M-NB1 showed a peak at 480 nm, after excitation at 350 nm, whereas the fluorescence emission spectrum of the *E. coli* produced A1M-WT had a maximum at 430 nm. Moreover, the 480 nm peak of A1M-NB1 was more than tenfold higher than that of A1M-WT (Figure 3B). This suggests a higher amount of chromophore, but the different profile may also indicate that the identities of the involved compounds differ from what is reported for *E. coli* produced A1M-WT (Kwasek et al., 2007) and human urine A1M (Berggård et al., 1999). The tryptophan fluorescence (Figure 3C), resulting from excitation at 290 nm, was less intense, but paralleled that of *E. coli* produced A1M-WT, suggesting a similar location of the tryptophan residues in the 3D-structure near the bottom of the lipocalin pocket, i.e., a correct folding of A1M-NB1. The fluorescence spectra taken together thus indicate that the plant-produced protein is correctly folded and has a capacity to form chromophoric modifications.

### Analyses of Biophysical Properties

**FIGURE 4:**
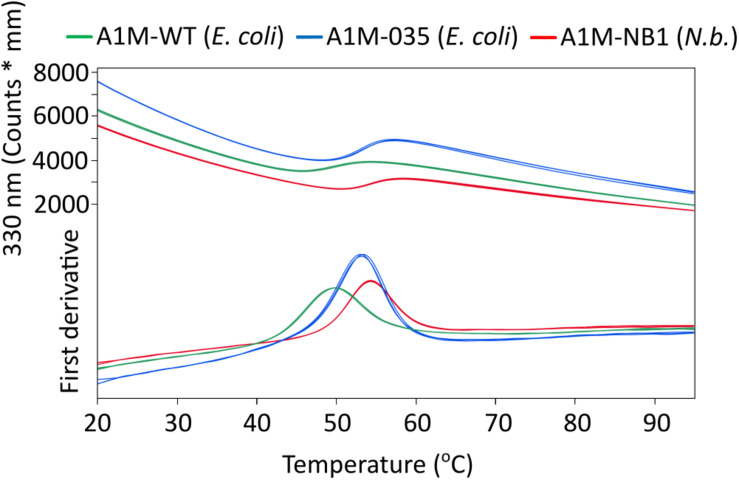
Differential scanning fluorimetry (DSF) analysis of rA1M temperature stability. Red: A1M-NB1, Blue: A1M-035 (*E. coli*), Green: A1M-WT (*E. coli*). Three replicates for each protein are shown (largely overlapping). *T*_m_ values were determined from the inflection points: A1M-NB1: 54.3 ± 0°C (*SD*, *n* = 3), A1M-035: 54.13 ± 0.15°C (*SD*, *n* = 3), A1M-WT: 49.8 ± 0.12°C (*SD*, *n* = 3).

As the reactivity of Cys34 is believed to be central to the function of A1M (Gunnarsson et al., 2017). Ellman’s test was employed to quantify the free sulfhydryl groups of surface accessible cysteine residues. The resulting value was ∼0.9 free surface cysteine residues per A1M-NB1 protein [0.92 ± 0.006 (*SD*, *n* = 3)], indicating the presence of a single free cysteine residue on the majority of the A1M-NB1 molecules, implying that the remaining two cysteine residues of A1M-NB1 are inaccessible or not in the reduced state. The value might be slightly underestimated as the bound chromophore may affect the absorption at 280 nm used for quantifying the protein. T_m_ values were determined using DSF: A1M-NB1: 54.3 ± 0°C (*SD*, *n* = 3), A1M-035: 54.13 ± 0.15°C (*SD*, *n* = 3), A1M-WT: 49.8 ± 0.12°C (*SD*, *n* = 3). The DSF analysis of A1M-NB1 indicated that it had a thermostability comparable to the control sample A1M-035, although the A1M-035 sample contained more dimer, as seen in Figure 2D, which may have affected the result for the control sample (Figure 4). This may be due to a longer storage time for that sample rather than a difference between the expression systems, as A1M can form dimers and aggregates over time (Åkerström et al., 2019). As expected A1M-WT was less thermostable than the two mutated rA1M variants. DLS analysis gave a radius of 2.9 ± 0.11 nm (*SD*, *n* = 6), comparable to previously reported values for *E. coli* produced rA1M (Åkerström et al., 2019).

### Heme Binding Test

**FIGURE 5:**
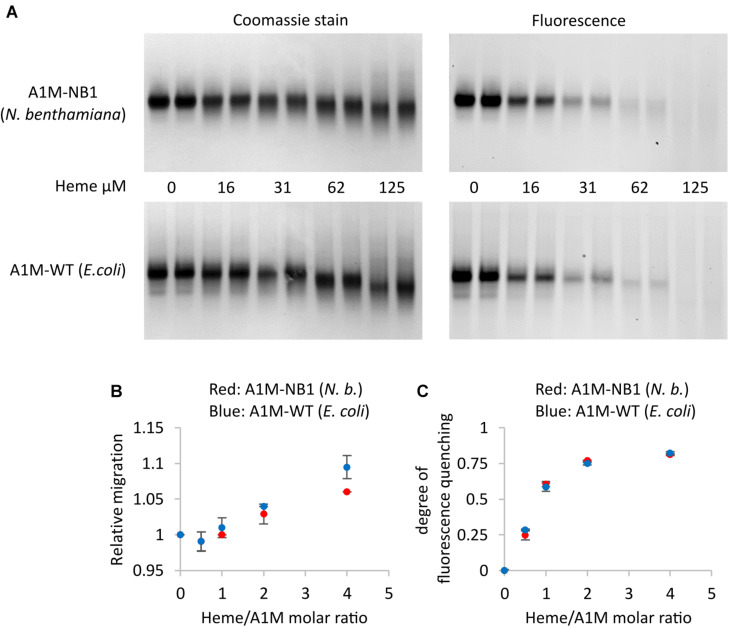
Heme binding of plant produced A1M-NB1 and *E. coli* produced A1M-WT. **(A)** 15 μg (∼34 μM) A1M-NB1 or A1M-WT were incubated with different concentrations of heme for 30 min at RT, separated on a 12% gel by native PAGE (two lanes per concentration), and analyzed by tryptophan fluorescence (fluorescence) and densitometry scanning after Coomassie staining (stain). Migration distance **(B)** and heme binding, measured as fluorescence quenching **(C)**, were plotted against the heme/rA1M molar ratio. Red: plant produced A1M-NB1, blue: *E. coli* produced A1M-WT. Means ± *SD* (*n* = 2) are shown. Digitalization was performed by using Image Lab software (Bio-Rad).

A well-documented property of A1M is heme binding (Karnaukhova et al., 2014). We investigated the heme binding of A1M-NB1 by several different methods. When analyzed on native PAGE and visualized using either staining or with its tryptophan fluorescence, addition of heme to A1M-NB1, as expected, resulted in a gel migration shift (Figures 5A,B) and fluorescence quenching (Figures 5A,C). When compared to the *E. coli* produced control sample (A1M-WT) the addition of heme seemed to have an identical effect on the fluorescence (Figures 5A,C), with a rapid increase in quenching as heme:rA1M molar ratio was increased to 2:1. Addition of heme increased the migration of both A1M-variants, although A1M-NB1 was somewhat less affected than A1M-WT (Figures 5A,B).

**FIGURE 6:**
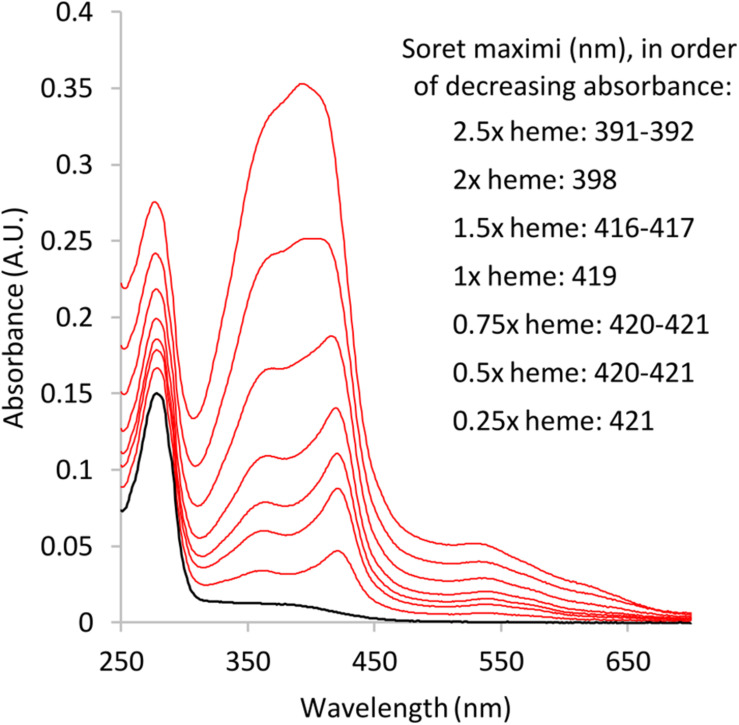
Absorbance spectra of A1M-NB1-heme mixtures after ∼2.5 h incubation. Black: A1M-NB1 spectrum without heme, ∼0.1 mg/mL. Red: Added heme/A1M-NB1 molar ratio: 2.5, 2, 1.5, 1, 0.75, 0.5, and 0.25, in order from max absorbance. Heme concentration is based on absorbance in the DMSO stock solution. Buffer: 20 mM Tris-HCl pH 8.0, 150 mM NaCl. Final DMSO concentration was <0.3% for all samples. The added heme concentrations should be seen as approximate for dissolved heme, due to the low stability of free heme in aqueous buffers.

The results from absorbance spectroscopy also support heme binding of A1M-NB1 (Figure 6). A main peak was detected around 422 nm at low heme concentrations, which shifted to shorter wavelengths at higher heme to A1M-NB1 ratios. At ratios above ∼1.5, the spectra appeared to be increasingly impacted by the absorbance of free heme, probably indicating increasing concentrations of unbound heme, which would be in line with previous results (Åkerström et al., 2019), though the instability of heme in the solution could also be a factor. The spectra were similar to those previously published for *E. coli*-produced A1M-WT and A1M-035 (Åkerström et al., 2019). A minor difference may be a more pronounced minor peak around 360 nm for A1M-NB1.

### Cell Protection Assay

**FIGURE 7:**
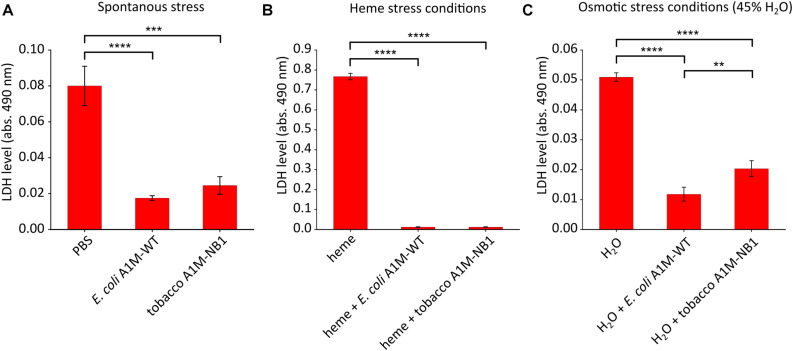
Cell protection assay. A1M-NB1 protects RBC’s against spontaneous, heme- and osmosis-induced hemolysis similar to the *E. coli* produced A1M-WT. Hemolysis, measured as LDH-release, after 2 h of incubation of washed RBCs with *E. coli* produced A1M-WT or A1M-NB1 (220 μg/mL) after spontaneous **(A)**, heme-induced (30 μM heme) **(B)** or osmotic-induced stress (H_2_O added to 45%) **(C)**. Values are presented as means ± *SD* (*n* = 3). Differences in groups were analyzed using a one-way ANOVA with post hoc Tukey’s **(A–C)**. **p < 0.01, ***p < 0.001, ****p < 0.0001.

A1M was recently shown to protect human adult- and fetal-RBC’s as well as mouse RBCs against cell death (Kristiansson et al., 2020a). The RBC protection assay was used here in order to evaluate the effect of plant-produced A1M-NB1 on cell protection. RBC stability was examined during spontaneous stress (stress from incubation and change from plasma to PBS), and after exposure to heme (30 μM) resulting in heme-induced stress, and addition of water (to 45%) resulting in osmotic stress. LDH leakage from the RBCs was used as a marker of hemolysis, since LDH leaks out of the RBCs to the surrounding media when cells lyse (Kato et al., 2006). A significant reduction in LDH leakage was observed after addition of A1M-NB1 during infliction of spontaneous (∼70%, Figure 7A), heme-induced (∼95%, Figure 7B) and osmotic stress (∼60%, Figure 7C). The protective effect of A1M-NB1 was similar to the *E. coli* produced A1M-WT, with the exception of having a slightly lower protection ability during the osmotic stress conditions (∼75% for A1M-WT compared to ∼60% for A1M-NB1).

## Discussion

We have shown in this study that a functional human rA1M variant, A1M-NB1, with two amino acid substitutions and an N-terminal His-tag, could be expressed in *N. benthamiana*, with a yield of ∼50 mg/kg (fresh leaf weight) purified protein. Two additional variants, targeted to the ER or for secretion (A1M-NB2 and A1M-NB3), were also expressed, without apparent proteolytic degradation, and appeared to be glycosylated as expected. These variants were however not tested for function in this study since we focused primarily on the cytosolic A1M-NB1, due to the apparent higher yield and homogeneity in the initial SDS-PAGE analysis. The purified A1M-NB1 protein appeared correctly folded, displayed a free surface cysteine residue and the presence of tightly bound chromophores, all typical structural features of plasma and urine A1M as well as of *E. coli*-produced recombinant A1M (Ekström and Berggård, 1977; Åkerstrom et al., 2000; Kwasek et al., 2007; Åkerström and Gram, 2014). Moreover, A1M-NB1 was shown to bind free heme and protect RBCs from stress-induced cell death, both previously described functions of plasma A1M and recombinant A1M expressed from *E. coli* cultures (Karnaukhova et al., 2014; Kristiansson et al., 2020a).

The physicochemical data collected for the A1M-NB1 protein indicate that it was correctly folded. The obtained values from DLS and thermostability and elution behaviour on the size exclusion chromatography are close to what has been reported for *E. coli*-produced A1M-035 (Åkerström et al., 2019). The results from Ellman’s test indicate the presence of a single surface accessible free cysteine residue on A1M-NB1, which would be expected on a functional, folded A1M protein (Ekström and Berggård, 1977). Furthermore, the shift in mobility between non-reduced and reduced SDS-PAGE for plant produced rA1M-NB1 (Figure 2D) suggests that an internal disulfide bridge was present at the same location as in human urine A1M (Mendez et al., 1982) and *E. coli* A1M-035 (Åkerström et al., 2019). This was however not so clear on some gels, which might indicate that a minor fraction lacking correct disulfide bridge formation was also present. If this was so, it did not appear to have a detrimental effect on the melting point of the protein, however. As A1M-NB1 was not targeted to the secretory pathway, generally associated with oxidative folding (Sevier and Kaiser, 2002), the indication of a disulfide bond might be surprising, though it could perhaps have formed after isolation in the non-reducing environment after the IMAC purification step. Reducing agent was included in the initial extraction steps to protect the exposed Cys34 residue, which may also have had an effect on disulfide bridge formation. The A1M-NB2 and A1M-NB3 showed a similar shift in size between reduced and non-reduced as the A1M-NB1 on the SDS-PAGE (Supplementary Figure 1B), suggesting that these proteins, expected to be subjected to oxidative folding, similarly featured a disulfide bond. Here too, the reducing agent DTT was used during the extraction procedure, however. The size exclusion chromatography of A1M-NB1 showed a distribution of A1M between monomers, dimers and multimer aggregates, a typical behavior of correctly folded human A1M (Ekström and Berggård, 1977). Finally, the tryptophan fluorescence spectrum of A1M-NB1 (Figure 3C) suggests a similar three-dimensional location of the tryptophan residues as in *E. coli*-produced A1M-WT (Kwasek et al., 2007; Åkerström et al., 2019).

Yellow-brown chromophores were bound to the purified A1M-NB1, as observed visually and by absorbance and fluorescence spectroscopy. The identities of the bound substances were not determined, but the spectral analyses revealed both similarities and differences when compared to human native A1M and *E. coli*-produced A1M-WT (Åkerström et al., 1995; Kwasek et al., 2007). A1M from the three sources display a heterogeneous absorbance of UV-light between 300 and 400 nm. Compared to that of the bacterially produced A1M-WT (Figure 3A), the plant-produced A1M-NB1 absorbance spectrum was more reminiscent of A1M isolated from human urine or mammalian- or insect cell culture (Åkerström et al., 1995), but with a more distinct shoulder, or plateau, at 390–400 nm. A1M-NB2 and A1M-NB3 also displayed absorbance spectra consistent with chromophore modifications, suggesting that the process also occurred in the ER and extracellular environment. The fluorescence spectrum of A1M-NB1 similarly showed a higher amount of chromophore, and positioning of the chromophore in the expected protein region. The formation of A1M chromophores, i.e., covalent side-group modifications, has been speculated to be a result of the pseudoenzymatic radical scavenging activity of the protein (Sala et al., 2004; Åkerström et al., 2007; Åkerström and Gram, 2014), which results in covalent trapping of free radical species. The higher level of bound chromophore seen in the plant-produced rA1M compared to the *E. coli*-produced rA1M could possibly be explained by a correct folding of plant rA1M in the cellular compartments, whereas bacterially produced rA1M forms precipitates (inclusion bodies) immediately after translation of the A1M-peptide. Therefore, A1M-NB1 may obtain radical scavenging activity already in the leaf cells of *N. benthamiana* allowing trapping of cellular radicals, while bacterially produced rA1M will not become enzymatically active until it is refolded *in vitro* later in the purification process. However, if the chromophoric additions indeed are a result of the covalent radical trapping mechanism, the higher level of bound chromophore may also imply a consumption of the potential radical binding sites, estimated to be at least 3 per A1M protein (Åkerström et al., 2007), and thus a reduced radical binding capacity of the A1M-NB1. The qualitative differences in the observed spectra of plant rA1M and human native A1M (Åkerström et al., 1995) also indicate that different chromophores attach to the protein, reflecting the availability of free radical compounds in the different biological systems.

The purified, plant-produced, A1M-NB1 was shown to bind heme in this study. Several techniques can be used to investigate heme binding by proteins (Comer and Zhang, 2018). Binding of heme to a protein is known to, among other effects, affect the Soret peak of the heme absorbance spectrum, quench fluorescence of nearby fluorophores, such as tryptophans of the interacting protein, and affect the mobility in native PAGE and SEC due to resulting changes in the heme-binding proteins (Comer and Zhang, 2018). We have previously employed different methods to show binding of heme to human plasma A1M (Allhorn et al., 2002) and *E. coli*-produced rA1M (Karnaukhova et al., 2014; Rutardottir et al., 2016). In this work, we have used spectroscopy, fluorescence quenching and electrophoretic migration shift to show that the heme binding of A1M-NB1 was very similar to that of *E. coli*-produced rA1M. Quenching of tryptophan has been useful for analysis of ligand binding to lipocalins since a conserved tryptophan residue is located inside the pocket of lipocalins, including A1M (Breustedt et al., 2006). The migration shift in native PAGE is most likely a result of a change in net charge of A1M upon heme binding. The small difference in migration shift in native PAGE between plant- and *E. coli*-produced rA1M may be due to the higher chromophore content of A1M-NB1, which could influence the net charge, or influence the effect of bound heme-groups on the net charge of the protein.

It was recently shown that rA1M produced in *E. coli* could protect RBCs from hemolysis induced by various forms of stress (Kristiansson et al., 2020a). Although the exact protection mechanisms are not known, it was speculated that the enzymatic activities of A1M, i.e., reduction, heme binding and radical binding, are involved to various degrees depending on the form of stress inflicted upon the cells. For example, the heme binding of A1M is obviously of major importance when the protein protects RBCs from heme-induced hemolysis, but it is difficult to attribute the protection against osmosis-induced and spontaneous hemolysis solely to the heme-binding capacity of A1M, but rather a combination of heme- and radical-binding and reductase activities. In this work, we could show that the A1M-NB1 expressed in *N. benthamiana* leaves also could protect RBCs from different forms of stress (Figure 7). This suggests that A1M-NB1 has a similar arsenal of enzymatic mechanisms as *E. coli*-produced A1M.

Two glycosylated versions of rA1M (NB2 and NB3) were included in this study, as native A1M carries several glycosylation motifs (Wester et al., 2000), which have been suggested to have a positive effect on the stability and solubility of native A1M (Åkerström et al., 2019). The plant N-glycosylation machinery works in a similar fashion as the mammalian, and produces a similar glycosylation by processing the protein first in the ER and then in the Golgi apparatus, and could thus serve as a substitute for the mammalian glycosylation of A1M, though potentially immunogenic plant specific glycan motifs might be added in the Golgi (Strasser, 2016; Rozov et al., 2018). The A1M-NB2 was designed for ER targeting for that reason, although an expected drawback might be faster blood-stream clearance (Ko et al., 2003; Rozov et al., 2018) while the glycosylation of secreted A1M-NB3 may contain plant specific motifs, and might require “glycoengineered” plants for safe production (Rozov et al., 2018; Schoberer and Strasser, 2018). The results of the rA1M deglycosylation test using PNGase F, which showed an apparent full deglycosylation of the A1M-NB2 variant (Supplementary Figure 1), and only partial deglycosylation of A1M-NB3 (Supplementary Figure 1), is in line with these expectations, as PNGase is not capable of cleaving the 1–3 fucose commonly found on plant N-linked oligosaccarides (Tretter et al., 1991). The result does not rule out the involvement of other possible posttranslational modification on A1M-NB3 however, such as O-glycosylation. The presence of N-glycosylation motifs produced by the plant did not appear to have any clear positive effect on the accumulation level of A1M-NB2 and A1M-NB3 when compared to the mutated A1M-NB1 variant. Further work would be needed to determine if the plant-produced glycosylation improves the stability and solubility of rA1M and how its effect compares to that of native mammalian glycosylation.

The plant expression system offers several advantages for the production of rA1M, compared to *E. coli expression*. Unlike rA1M previously produced in bacteria, where the protein is isolated from inclusion bodies (Kwasek et al., 2007), the A1M-NB1 does not require refolding, which should reduce the losses during isolation. As has been discussed above, the plant could also offer qualitative advantages, such as the possibility of glycosylation and increased safety due to lack of, or at least lower levels of endotoxins of microbial origin. The higher degree of saturation with chromophore compared to the *E. coli* produced rA1M could however be a drawback, as it might indicate a consumption of potential radical trapping sites, as explained earlier in the discussion. Future development of *N. benthamiana* expression of rA1M for therapeutic use should therefore involve methods to limit chromophore formation. While the syringe based agroinfiltration technique used here is limited in scalability, other techniques for transient expression such as vacuum infiltration (Chen et al., 2013) and agrospray (Azhakanandam et al., 2007; Hahn et al., 2015) could potentially be used for larger scale protein production.

In conclusion, the study showed that expression of functional rA1M was possible in *N. benthamiana.* The characterization efforts of the purified A1M-NB1 revealed that the protein had the expected functional and structural properties. *N. benthamiana* could therefore be a future option for production of rA1M for pharmaceutical purposes, although further development and optimization would be required. Additional variants of rA1M targeted to the secretory pathway were also expressed, but were not further investigated in this study, although that approach may offer future possibilities for an improved product. The plant-produced A1M-NB1 appeared to have a similar, but not identical, chromophore profile, when compared to A1M from other sources, which could be useful when delineating the identity of the compounds involved.

## Data Availability Statement

The raw data supporting the conclusions of this article will be made available by the authors, without undue reservation.

## Ethics Statement

The studies involving human participants were reviewed and approved by the Ethical Review Board in Lund (Permission no. 2015/801). The patients/participants provided their written informed consent to participate in this study.

## Author Contributions

L-HZ and BÅ initiated the project. MC performed the majority of cloning, protein production and purification, with input and contributions from BÅ and SK. Protein characterization and analysis was performed by MC, AK, and JB with input from BÅ and LB. MC, BÅ, and L-HZ wrote the manuscript with contribution and input from the other authors. All authors contributed to the article and approved the submitted version.

## Conflict of Interest

The authors declare that the research was conducted in the absence of any commercial or financial relationships that could be construed as a potential conflict of interest.
