# Supplementary material for: Expression, Purification and Initial Characterization of Functional α1-Microglobulin (A1M) in Nicotiana benthamiana
Source: Front Plant Sci. 2020 Dec 8;11:593773. doi: 10.3389/fpls.2020.593773 (PMC7752767; doi:10.3389/fpls.2020.593773)
Supplement: Supplementary file 1 [file Data_Sheet_1.pdf]

Supplementary data

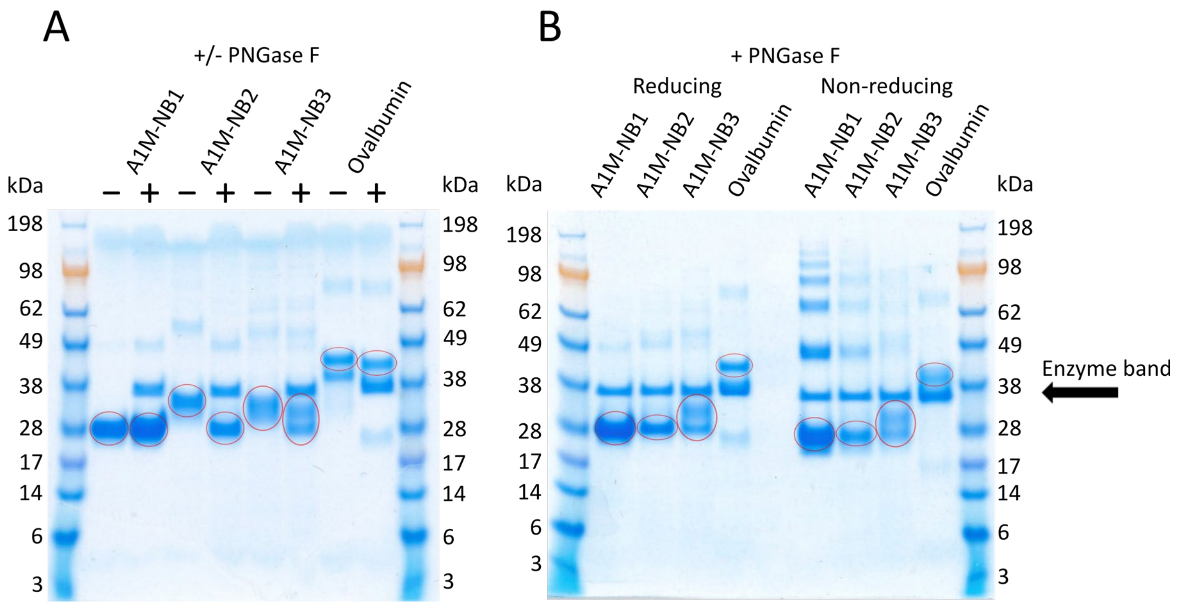

Supplementary figure 1. Enzymatic deglycosylation of rA1M. Plant produced rA1M targeted to cytosol (A1M-NB1), ER (A1M-NB2) and extracellular space (A1M-NB3) were treated with PNGase F for removal of N-glycosylation. Ovalbumin was included as a control sample. Main bands of interest for each protein, as previously identified by western blot (Fig. 1), are marked by red circles (other bands include the presumed enzyme and A1M dimer and aggregates/multimers). Note that the amounts of multimers and aggregates (assumed to constitute most of the bands above 38 kDa for the non-reduced samples) should not be considered representative of the isolated A1M samples as they were denatured prior to incubation with the enzyme, and aggregates may have formed at that time. **A:** rA1M reactions with or without PNGase F enzyme (+/-). A decrease in molecular weight is seen for A1M-NB2 and A1M-NB3, consistent with deglycosylation, A1M-NB3 displays heterogeneity after the treatment and may not be fully deglycosylated. **B:** Deglycosylation and SDS-PAGE performed under reducing or non-reducing conditions. A similar difference in apparent molecular weight is seen between the two conditions for the three plant produced A1M variants, indicating a similar disulfur bond conformation.

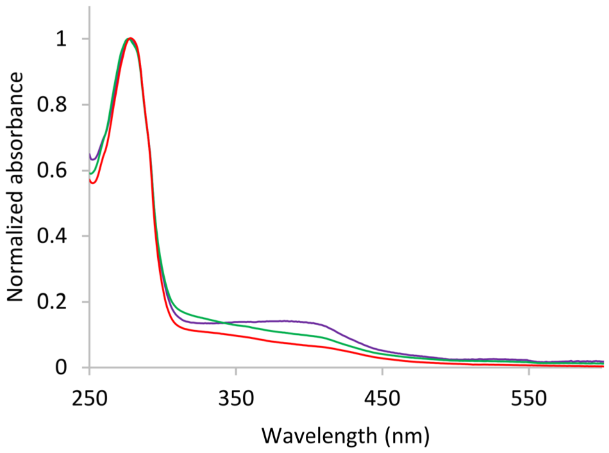

Supplementary figure 2. Normalized absorbance spectra (ratio of 277 nm abs) of partially purified (post IMAC and diafiltration) rA1M. Red: A1M-NB1, purple: A1M-NB2, green: A1M-NB3. All rA1M variants displayed a visible yellow-brown

color. A1M-NB1 was extracted and purified at a larger scale than the other samples so may be less pure than the other samples shown here.

Sequences

A1M-NB1

Nucleotide sequence

(start and stop codons marked in green and red)

AGTCTTAATTAAGCCACCATGCATCATCACCACCATCACCATCACGATGATGATGATAAGGGCCCTGTTCCTAC
TCCTCCTGATAACATTCAGGTGCAAGAGAACTTCGACATCAGCCGGATCTACGGCAAGTGGTACAACCTTGCT
ATCGGCTCTACTTGCCCGTGGCTGAAGAAAATCATGGACAGGATGACCGTGAGCACCCTTGTTCTTGGTGAAG
GTGCTACTGAGGCCGAGATCTCTATGACTTCTACTAGATGGCGTAAGGGCGTGTGCGAAGAAACTTCTGGTGC
TTACGAAAAGACCGACACCGACGGCAAGTTCCTCTACCATAAGAGCAAGTGGGACATCACCATGGAAAGCTA
CGTGGTGCACACCAACTACGACGAGTACGCTATTTTCCTGACCAAGAAATTCAGCCGGCATCACGGTCCTACC
ATTACCGCTAAGCTTTATGGTAGGGCTCCACAGCTTAGGGAAACCCTGCTTCAGGATTTCAGAGTGGTTGCTC
AAGGTGTGGGTATCCCTGAGGATTCCATTTTCACCATGGCTGATAGGGGCGAGTGTGTTCCAGGTGAACAAG
AACCTGAGCCTATTCTGATCCCAAGGTAGTAGCCTAGGCGGC

Amino acid sequence

(non-native elements and modified residues (N->D) in blue, expected posttranslational removal of sections in parenthesis)

(M)HHHHHHHHDDDDKGPVPTPPDNIQVQENFDISRIYGKWYNLAIGSTCPWLKKIMDRMTVSTLVLGEGATEA
EISMTSTRWRKGVCEETSGAYEKTDTDGKFLYHKSKWDITMESYVVHTNYDEYAIFLTKKFSRHHGPTITAKLYGRA
PQLRETLLQDFRVVAQGVGIPEDSIFTMADRGECVPGEQEPEPILIPR--

A1M-NB2

Nucleotide sequence

(start and stop codons marked in green and red)

AGTCTTAATTAAGCCACCATGGGCTTCGTGCTGTTCTCTCAGTTGCCTTCTTTCCTGCTTGTGTCTACCCTTCTGC
TGTTCCTGGTGATCTCTCATTCTTGCAGGGCTCATCATCACCACCATCACCATCACGATGATGATGATAAGGGC
CCTGTTCCTACTCCTCCTGATAACATTCAGGTGCAAGAGAACTTCAACATCAGCCGGATCTACGGCAAGTGGTA
CAACCTTGCTATCGGCTCTACTTGCCCGTGGCTGAAGAAAATCATGGACAGGATGACCGTGAGCACCCTTGTT
CTTGGTGAAGGTGCTACTGAGGCCGAGATCTCTATGACTTCTACTAGATGGCGTAAGGGCGTGTGCGAAGAA
ACTTCTGGTGCTTACGAAAAGACCGACACCGACGGCAAGTTCCTCTACCATAAGAGCAAGTGGAACATCACCA
TGGAAAGCTACGTGGTGCACACCAACTACGACGAGTACGCTATTTTCCTGACCAAGAAATTCAGCCGGCATCA
CGGTCCTACCATTACCGCTAAGCTTTATGGTAGGGCTCCACAGCTTAGGGAAACCCTGCTTCAGGATTTCAGA
GTGGTTGCTCAAGGTGTGGGTATCCCTGAGGATTCCATTTTCACCATGGCTGATAGGGGCGAGTGTGTTCCAG
GTGAACAAGAACCTGAGCCTATTCTGATCCCAAGGGGCTCTAAGGATGAGCTGTAGTAGCCTAGGCGGC

Amino acid sequence

(non-native elements in blue and purple, expected posttranslational removal of sections in parenthesis)

(MGFVLFSQLPSFLLVSTLLLFLVISHSCRA)HHHHHHHHDDDDKGPVPTPPDNIQVQENFNISRIYGKWYNLAIGST
CPWLKKIMDRMTVSTLVLGEGATEAEISMTSTRWRKGVCEETSGAYEKTDTDGKFLYHKSKWNITMESYVVHTNY
DEYAIFLTKKFSRHHGPTITAKLYGRAPQLRETLLQDFRVVAQGVGIPEDSIFTMADRGECVPGEQEPEPILIPRGSKD
EL--

A1M-NB3

Nucleotide sequence

(start and stop codons marked in green and red)

AGTCTTAATTAAGCCACCATGGGCTTCGTGCTGTTCTCTCAGTTGCCTTCTTTCCTGCTTGTGTCTACCCTTCTGC
TGTTCCTGGTGATCTCTCATTCTTGCAGGGCTCATCATCACCACCATCACCATCACGATGATGATGATAAGGGC
CCTGTTCCTACTCCTCCTGATAACATTCAGGTGCAAGAGAACTTCAACATCAGCCGGATCTACGGCAAGTGGTA
CAACCTTGCTATCGGCTCTACTTGCCCGTGGCTGAAGAAAATCATGGACAGGATGACCGTGAGCACCCTTGTT
CTTGGTGAAGGTGCTACTGAGGCCGAGATCTCTATGACTTCTACTAGATGGCGTAAGGGCGTGTGCGAAGAA
ACTTCTGGTGCTTACGAAAAGACCGACACCGACGGCAAGTTCCTCTACCATAAGAGCAAGTGGAACATCACCA
TGGAAAGCTACGTGGTGCACACCAACTACGACGAGTACGCTATTTTCCTGACCAAGAAATTCAGCCGGCATCA
CGGTCCTACCATTACCGCTAAGCTTTATGGTAGGGCTCCACAGCTTAGGGAAACCCTGCTTCAGGATTTCAGA
GTGGTTGCTCAAGGTGTGGGTATCCCTGAGGATTCCATTTTCACCATGGCTGATAGGGGCGAGTGTGTTCCAG
GTGAACAAGAACCTGAGCCTATTCTGATCCCAAGGTAGTAGCCTAGGCGGC

Amino acid sequence

(non-native elements in blue and purple, expected posttranslational removal of sections in parenthesis)

(MGFVLFSQLPSFLLVSTLLLFLVISHSCRA)HHHHHHHHDDDDKGPVPTPPDNIQVQENFNISRIYGKWYNLAIGST
CPWLKKIMDRMTVSTLVLGEGATEAEISMTSTRWRKGVCEETSGAYEKTDTDGKFLYHKSKWNITMESYVVHTNY
DEYAIFLTKKFSRHHGPTITAKLYGRAPQLRETLLQDFRVVAQGVGIPEDSIFTMADRGECVPGEQEPEPILIPR—

Vector specific primer (pJL-TRBO)

Forward: GATCTTACAGTATCACTACTCCATC

Reverse: CCGTTATTTATTATGCATCTTGACTACC
